# Research on an Infrared Multi-Target Saliency Detection Algorithm under Sky Background Conditions

**DOI:** 10.3390/s20020459

**Published:** 2020-01-14

**Authors:** Shaosheng Dai, Dongyang Li

**Affiliations:** College of Communication and Information Engineering, Chongqing University of Posts and Telecommunications, Chongqing 400065, China; daiss@cqupt.edu.cn

**Keywords:** sky background, infrared multi-target, multi-scale top-hat, multi-scale saliency fusion

## Abstract

Aiming at solving the problem of incomplete saliency detection and unclear boundaries in infrared multi-target images with different target sizes and low signal-to-noise ratio under sky background conditions, this paper proposes a saliency detection method for multiple targets based on multi-saliency detection. The multiple target areas of the infrared image are mainly bright and the background areas are dark. Combining with the multi-scale top hat (Top-hat) transformation, the image is firstly corroded and expanded to extract the subtraction of light and shade parts and reconstruct the image to reduce the interference of sky blurred background noise. Then the image obtained by a multi-scale Top-hat transformation is transformed from the time domain to the frequency domain, and the spectral residuals and phase spectrum are extracted directly to obtain two kinds of image saliency maps by multi-scale Gauss filtering reconstruction, respectively. On the other hand, the quaternion features are extracted directly to transform the phase spectrum, and then the phase spectrum is reconstructed to obtain one kind of image saliency map by the Gauss filtering. Finally, the above three saliency maps are fused to complete the saliency detection of infrared images. The test results show that after the experimental analysis of infrared video photographs and the comparative analysis of Receiver Operating Characteristic (ROC) curve and Area Under the Curve (AUC) index, the infrared image saliency map generated by this method has clear target details and good background suppression effect, and the AUC index performance is good, reaching over 99%. It effectively improves the multi-target saliency detection effect of the infrared image under the sky background and is beneficial to subsequent detection and tracking of image targets.

## 1. Introduction

In recent years, with the development of infrared technology and computer processing technology, infrared image processing has been widely used in military and civil fields [1]. Because of the low signal-to-noise ratio of infrared image and the weak infrared target in the sky background, the subsequent detection and tracking of the target in the sky are affected, so the detection of infrared targets becomes the key technology of infrared applications in the sky [2,3]. Image saliency is used in computer vision processing, and can highlight image target information. Therefore, using saliency detection for infrared image under sky background can make infrared targets more obvious, which is conducive to target detection. However, image target saliency detection mainly focuses on a single type of target [4], and normally a complex situation of multiple types of targets exists in the sky, so detection methods for multiple targets in infrared images need to be studied.

At present, saliency methods are mainly divided into two categories, one is bottom-up saliency detection based on the underlying features of an image, the other is top-down saliency detection based on the prior information of the target. For example, the early classic saliency model—the ITTI model of Itti and Koch et al. [5]—extracts three features (color, brightness and central peripheral difference) from an image to calculate the characteristic saliency map. The Winner Take All (WTA) mechanism is used to label the salient objects of the image, which mainly uses the bottom features, but the calculation speed is too slow for practical use. The overall resolution of the salient map is low and the salient target is blurred. Then, based on the ITTI model, the Graph Based Visual Saliency (GBVS) model was proposed by Harel et al. [6]. A Markov random field is used to calculate salient features, and its stable state is used to obtain salient maps. However, with the increase of image resolution, the amount of computation will continue to be too large, and the effect will also be reduced. With the continuous development of information theory, Hou [7] et al. put forward a more rapid saliency calculation model—the Spectral Residual (SR) model—from the perspective of frequency. The SR model of a spectrum residual is mainly based on the Fourier transform and spectrum residual information. After inverse Fourier transforms, saliency maps are obtained and the target is highlighted, but too much edge information is lost. Later, the saliency models Achanta (AC) [8] and Frequency-Tuned (FT) [9] of Achanta et al. appeared. AC models are all based on local contrast. The saliency of target centers is insufficient and not highlighted overall. FT models are based on global contrast, are faster, and the saliency of target centers is improved. Gou et al. [10] also considered the phase spectrum, using the Fourier function of the quaternion, using the brightness, chroma and motion characteristics of the image to obtain the phase spectrum and get the salient image. Li et al. [11] then weighted the model based on its features to improve the visual quality of the model. Xiao et al. [12] also proposed the saliency detection of a multi-scale phase spectrum, which can quickly generate saliency maps in the complex background and adapt to multi-target detection, but the image target is not overall highlighted. To fully highlight the saliency of the target, a saliency model is constructed by combining various methods. To better highlight the features of infrared targets in complex environments, Liu et al. [13] proposed an infrared image saliency detection method that fuses local and global features. The target area is highlighted as a whole, the edges are clear, and the background suppression effect is good. However, for very blurred infrared images, the target region is incomplete. Liu et al. [14] also used the top-down saliency detection method and proposed a saliency detection method based on regional covariance and target degree. The method combines Conditional Random Field (CRF) and dictionary learning. The goal is highlighted overall, and the background is suppressed, but the top-down method requires not only a lot of training and testing, but also artificial supervision training, making the workload very large. Jia et al. [15] proposed a multi-scale saliency detection method. Firstly, the image is structured and preprocessed. Then different saliency maps were obtained by using color space distribution, super-pixel segmentation and block scale to get the final saliency map by CRF fusion. This method has a good effect, but it needs a lot of training to obtain the model and training is excessive.

In this paper, a multi-target saliency detection method based on the fusion of multiple saliency detection is proposed. It is considered that the main problems of the infrared image in sky background situations are the dim and blurred sky background, low signal-to-noise ratio and the existence of dim and small targets. Firstly, the Top-hat transform improved by the multi-scale is used to preprocess the original infrared image, enhance the target in the original image, suppress the interference of background noise, and obtain the image with multiple targets highlighted. Then, according to the parameters in the frequency domain of the acquired image pixels, three improved salient maps, namely, spectrum residual, phase spectrum and Fourier transform, are fused to generate the salient maps of the final infrared target. After a series of real infrared image experiments and comparison with various classic salience models, the algorithm can detect multiple types of targets simultaneously, the saliency detection of the targets is better, the targets are complete and the edges of the targets are clear.

## 2. Main Algorithm

### 2.1. Multi-Scale Top-Hat

In a sky background, the infrared target is often on the cloud layer or hidden in the background. To better retain only the target pixel information and reduce background interference, infrared images need to be processed by background suppression to remove a large amount of background noise. Top-hat light and dark processing transformation can suppress the background well and retain the target, but the shape and size of the target are different. To better preserve different targets, this paper uses a multi-scale Top-hat transform to process infrared images.

**Figure 1:**
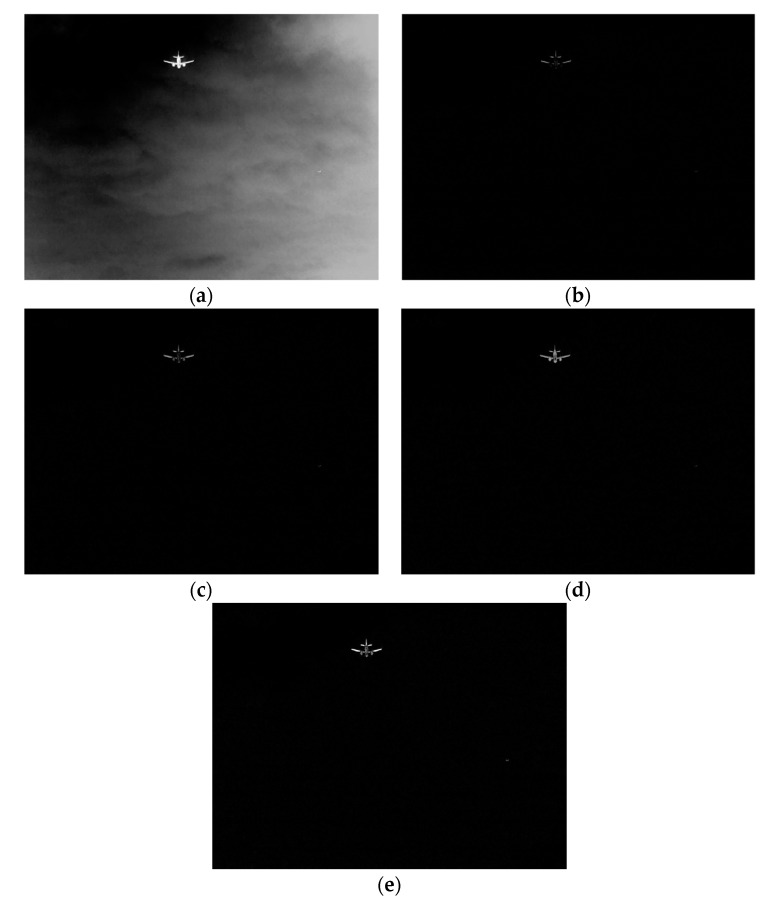
The result of a multi-scale Top-hat transform. (**a**) Enhanced image before Top-hat transform. (**b**) 3 × 3 Top-hat transform. (**c**) 5 × 5 Top-hat transform. (**d**) 7 × 7 Top-hat transform. (**e**) Image after multi-scale Top-hat transform.

Based on the traditional Top-hat transform, the structural element template is mainly used for morphological corrosion and expansion to extract the target. The background of the infrared image is complex. The complex background noise can be removed by Top-hat transform, and the target can be well preserved. The specific principle of the algorithm is that the original infrared gray-scale image is first eroded and then expanded to obtain the open-operation image. The open-operation image is subtracted from the original infrared gray image to extract the bright details of the image, which corresponds mainly to the image target part. Then the original infrared gray-scale image is first expanded and then eroded to obtain the close-operation image. The close-operation image is subtracted from the original infrared gray image to extract the dark details of the image, which mainly corresponds to the background of the image. This is calculated as follows:

(1) h=f−(fΘm)⊕m

(2) l=f−(f⊕m)Θm

where Θ is the symbol of corrosion operation, ⊕ is the symbol of expansion operation, m is the template of corrosion and expansion, f is the original infrared gray image, h is the bright area of the extracted image, l is the dark area of the extracted image. Finally, the light and dark areas extracted from the original infrared gray image are subtracted to obtain the final result after the Top-hat transformation calculated as follows:

(3) $$f_{r}=h-l$$

where $f_{r}$ is the image after Top-hat processing. To make the Top-hat transform retain both small and large targets when suppressing background, multi-scale Top-hat transform is used to select different sizes of square templates for image manipulation. Square templates with sizes of 3, 5 and 7 are mainly selected, and then images processed with different sizes of templates are fused. The calculation formula used for fusion is as follows:

(4) $$f_{t}=k\sum_{i}^{n}fuse_{N_{i}}f_{N_{i}}$$

(5) $$fuse_{N_{i}}=(n-i+1)/n$$

where n is the number of templates, i is the serial number corresponding to the template, $f_{N_{i}}$ is the image after Top-hat transform of the template with size N×N, k is the image gain control coefficient, $fuse_{N_{i}}$ is the fusion coefficient of the multi-size image, $f_{t}$ is the result of background suppression of multi-scale Top-hat transform. Through multi-scale Top-hat transformation, various objects of different sizes are retained, and the useless background information in the image is effectively suppressed, which reduces the background interference and is conducive to better detection of the target. The infrared image is suppressed by a multi-scale Top-hat change background as shown in Figure 1.

### 2.2. Multiple Saliency Detection Fusion

The size of the target in the image is different, there are some fragments and incompleteness in target detection, and the small and weak targets cannot be highlighted very well. To make the targets more prominent and fully highlight the target, it is necessary to make the small targets more obvious and the weak part of the target more completely prominent. Target saliency extraction can well highlight the weak targets in the image, which is conducive to small target detection and the overall prominence of large targets.

To comprehensively detect the saliency of an image, three saliency detection methods were used to fuse, which are the saliency detection based on spectrum residuals, the saliency detection based on phase spectrum and the saliency detection based on quaternion Fourier transform, respectively. The saliency extraction based on spectral residuals is to transform the image from the time domain to the frequency domain, calculate the spectrum and spectral residuals, and then filter to reconstruct the image. The abnormal part of the image is salient. The algorithm mainly extracts the background of the image, uses a smoothing filter to smooth the frequency spectrum of the frequency domain amplitude, and then removes the background to highlight the abnormal part of the image. The principle of the algorithm is to transform the image obtained from a multi-scale Top-hat transform to the frequency domain by Fourier transform and calculate the amplitude and phase in the frequency domain as follows:

(6) A(f)=|F[I(x)]|

(7) P(f)=φ(F[I(x)])

where F is Fourier transform, φ is phase extraction function, I(x) is multi-scale Top-hat transform image, A(f) is the amplitude, P(f) is the phase. Then, for ease of calculation and expansion of nuances, the amplitude spectrum of the image is obtained by the logarithm of amplitude A(f), and then the background spectrum is obtained by using the local average filter template to smooth the amplitude spectrum. The calculate these as follows:

(8) L(f)=log(A(f))

(9) $$V(f)=L(f)\times h_{n}(f)$$

(10) $$h_{n}(f)=\frac{1}{n^{2}}\left(\begin{array}{ccc} 1 & \ldots & 1\\ \vdots & \ddots & \vdots \\ 1 & \cdots & 1 \end{array}\right)$$

where L(f) is the amplitude spectrum, V(f) is the background spectrum, $h_{n}(f)$ is the local average filter template, n is the size of the template, size 3 is more appropriate for smoothing the details of the image better. Finally, the spectral residual is obtained by subtracting the background spectrum V(f) from the amplitude spectrum L(f), which can well represent the abnormal part of the image, including the target. The calculation is as follows:

(11) R(f)=L(f)−V(f)

where R(f) is the spectrum residual. Then the spectral residuals and phases are inversely transformed by Fourier transform, and the saliency map $S_{A}(x)$ is reconstructed by Gauss filtering, calculated as follows:

(12) $$S_{A}(x)=G(x)\times |F^{-1}[\exp\left\{R(f)+iP(f)\right\}]{|}^{2}$$

where i is an imaginary number unit, exp is an exponential function, $F^{-1}$ is an inverse Fourier transform, G(x) is a Gauss filtering function, $S_{A}(x)$ is a saliency detection map of spectrum residuals. However, the size of the target is different. To get the target of various sizes, the algorithm is improved by using multi-scale saliency detection, using several different size Gaussian filter templates, and using a maximum fusion algorithm for saliency detection of different scales. The scale fusion algorithm is expressed as follows:

(13) $$S_{Am}(x)=\max(S_{A_{g_{N}}}(x))$$

where $S_{Am}(x)$ is a multi-scale saliency detection map of spectrum residuals, $S_{A_{g_{N}}}(x)$ is a saliency map reconstructed by a Gauss filter template of N size, and the maximum value of N is 10. The final saliency map can be adapted to different sizes and shape targets, which can highlight the target well.

Because the amplitude information sometimes cannot fully represent the saliency of the target, it needs another detection supplement. Based on the saliency detection of the phase spectrum, the saliency information of the target is obtained mainly from the phase information. The principle of the algorithm is that the multi-scale Top-hat transformed gray image is first transformed into the phase spectrum in the frequency domain by Fourier transform, then the phase spectrum of the image is inversely transformed by Fourier transform, and the saliency map is obtained by Gauss filtering, calculated as follows:

(14) P(f)=φ(F[I(x)])

(15) $$S_{\varphi }(x)=G(x)\times |F^{-1}[\exp\{iP(f)\}]{|}^{2}$$

where i is the imaginary number unit, exp is the exponential function, F is the Fourier transform, $F^{-1}$ is the inverse Fourier transform, G(x) is the Gauss filter function, P(f) is the phase spectrum of the image, $S_{\varphi }(x)$ is the saliency detection image of the phase spectrum. Similarly, multi-scale saliency detection is used to improve the algorithm and adapt to target saliency detection of different sizes. The scale fusion algorithm is obtained as follows:

(16) $$S_{\varphi m}(x)=\max(S_{\varphi_{g_{N}}}(x))$$

where $S_{\varphi m}(x)$ is a multi-scale saliency detection map of phase spectrum, $S_{\varphi_{g_{N}}}(x)$ is a saliency map reconstructed by a Gauss filter template of N size, and the maximum value of N is 10.

After the above two kinds of saliency detection, the edge of the target is usually too blurred. To highlight the edge and detail information, the saliency map is improved by using quaternion Fourier saliency detection. The quaternion Fourier transform is based on the characteristics of human visual system, using four independent features to represent each image, and then quaternary features and phase spectrum are used to detect the saliency of the target, and the saliency of the target in the image is expressed in detail according to the characteristics of the human eye. Because of the need to use the color information of the image, the gray-scale image after multi-scale Top-hat transformation, first through 256 pseudo-color mappings, gets the Red Green Blue (RGB) image and then carries on the quaternary Fourier saliency detection. The principle of the algorithm is to extract the color features of the RGB image obtained from the mapping as follows:

(17) R(i)=r(i)−(g(i)+b(i))/2

(18) G(i)=g(i)−(r(i)+b(i))/2

(19) B(i)=b(i)−(r(i)+g(i))/2

(20) Y(i)=(r(i)+g(i))/2−|r(i)−g(i)|/2−b(i)

where r(i), g(i) and b(i) are the red, green and blue color features of the original RGB image obtained by pseudo-color mapping respectively. R(i), G(i), B(i) and Y(i) are the adjusted red, green, blue and yellow color features respectively. Then the binary features RG(i) and BY(i) are calculated, which correspond to the two neurons of human visual perception. The calculation is as follows:

(21) RG(i)=R(i)−G(i)

(22) BY(i)=B(i)−Y(i)

Other two-dimensional feature are the brightness feature and motion feature, which are I(i) and M(i). The brightness feature is obtained from the red, green and blue color features of the original RGB image obtained by pseudo-color mapping. The motion feature is obtained by the frame difference method according to the brightness difference between the two images. The necessary calculations are as follows:

(23) I(i)=(r(i)+g(i)+b(i))/3

(24) M(i)=|I(i)−I(i−1)|

where I(i) is the brightness feature and M(i) is the motion feature. Finally, a quaternion function is constructed. The phase spectrum is obtained by Fourier transform. The phase spectrum is filtered by inverse Fourier transform and Gauss filter to obtain the saliency detection map $S_{qp}(i)$ based on the quaternary Fourier transform calculated as follows:

(25) $$q(i)=M(i)+RG(i)\mu_{1}+BY(i)\mu_{2}+I(i)\mu_{3}$$

(26) $$S_{qp}(i)=G\times ||q^{\prime }(i)|{|}^{2}$$

where $\mu_{1}$, $\mu_{2}$ and $\mu_{3}$ are characteristic weight adjustment coefficients, G is Gauss filtering function, q(i) is quaternion characteristic function, $q^{\prime }(i)$ is the result of the phase spectrum of quaternion function obtained by Fourier transform and then by inverse Fourier transform.

**Figure 2:**
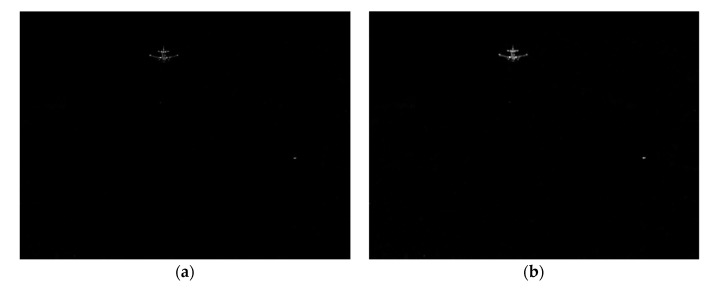

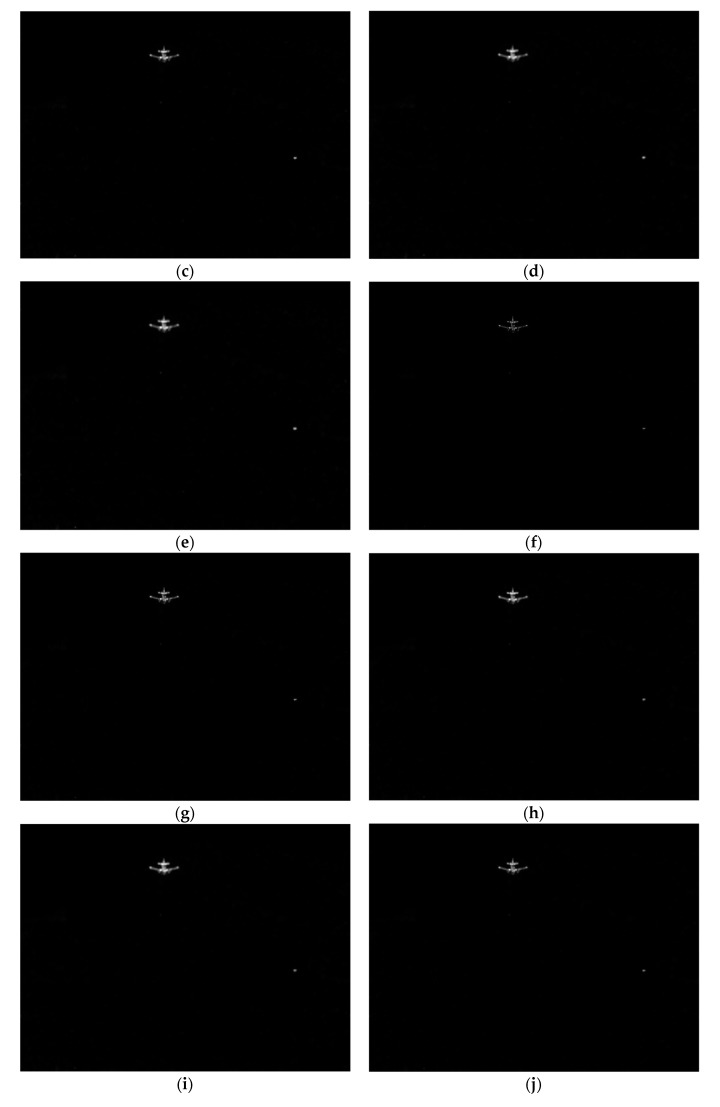

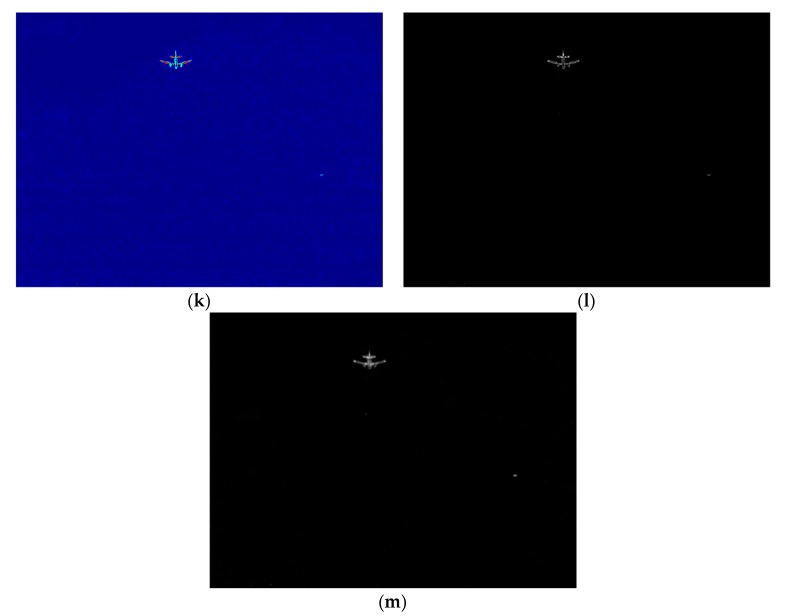
Multiple saliency detection results and fusion image. (**a**) 3 × 3 spectrum residual. (**b**) 5 × 5 spectrum residual. (**c**) 7 × 7 spectrum residual. (**d**) 10 × 10 spectrum residual. (**e**) Multi-scale spectrum residual method. (**f**) 3 × 3 phase spectrum. (**g**) 5 × 5 phase spectrum. (**h**) 7 × 7 phase spectrum. (**i**) 10 × 10 phase spectrum. (**j**) Multi-scale phase spectrum method. (**k**) The RGB image after multi-scale Top-hat transformation (**l**) Quaternion Fourier transform. (**m**) Fusion algorithm in this paper.

The saliency map based on the Fourier transform contains more detail information and edge information, and a more detailed saliency target, which makes up for the shortcomings of the two saliency detection methods mentioned above, but cannot highlight the saliency of the target as a whole. Finally, combining three saliency detection algorithms, a variety of saliency detection fusion is improved. Because the weights of the three detection algorithms are the same, they have their own shortcomings and advantages, so the method of equalization is used to fuse the three saliency detection results $S_{Am}(x)$, $S_{\varphi m}(x)$ and $S_{qp}(x)$. The fusion algorithm is calculated as follows:

(27) $$S_{fuse}(x)=\frac{S_{Am}(x)}{3}+\frac{S_{\varphi m}(x)}{3}+\frac{S_{qp}(x)}{3}$$

where $S_{Am}(x)$ is the multi-scale saliency detection map of spectrum residual, $S_{\varphi m}(x)$ is the multi-scale saliency detection map of the phase spectrum, $S_{qp}(x)$ is the saliency detection map of the quaternion Fourier transform, $S_{fuse}(x)$ is the fusion saliency map of three saliency detection. The gray-scale image after multi-scale Top-hat transformation is processed by a multi-scale saliency fusion method as shown in Figure 2. The final saliency map effect obtained by fusion is shown in Figure 2m. From the four graphs of Figure 2e,j,l,m, we can see that using the fusion algorithm in this paper, the saliency target is not only clear and the edge is obvious, but also the whole target is complete and undivided. The saliency effect of the infrared image is good.

## 3. Experimental Results and Analysis

To analyze the saliency detection results of infrared multi-target images by the proposed algorithm, the qualitative and quantitative analyses are carried out, respectively. Firstly, through qualitative analysis, the excellent performance of infrared target saliency detection results of this algorithm is evaluated through human visual sensory evaluation. To test the robustness of this algorithm, then this algorithm is analyzed quantitatively by comparing it with other classical salience detection algorithms. The algorithm simulation experiments are carried out on the same personal laptop computer. The computer hardware includes an Intel i3-3110M 2.3 GHz Central Processing Unit (CPU) core and 12 G running memory. The operating system used is Windows 7 64 bits, and the simulation software is Matrix Laboratory (MATLAB) R2016b.

### 3.1. Analysis of Our Algorithm

By listing the improvement effects of each step of this algorithm and observing its performance, we will be able to observe the effect of our algorithm better. In order to verify that the saliency detection algorithm in this paper can detect the saliency of targets of different sizes in the case of sky blurred background, and can highlight the small and weak targets hidden in the background, three types of infrared images under the background of sky clouds are adopted.

**Figure 3:**
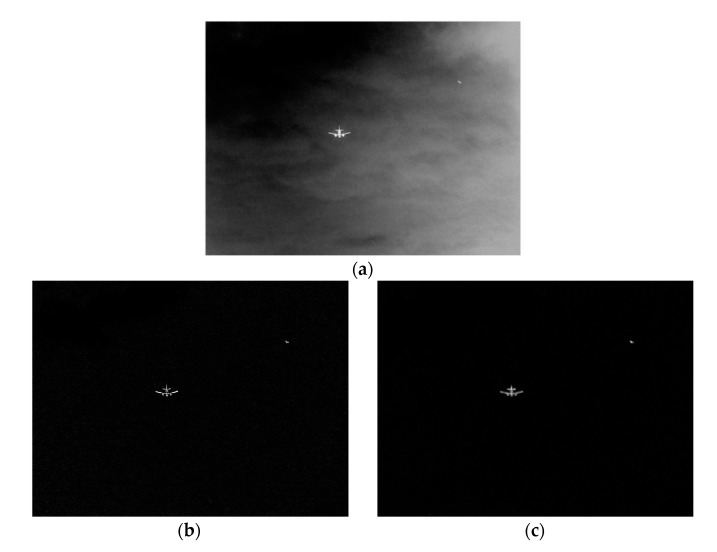
The saliency detection results of this algorithm for the first kind of infrared image. (**a**) The original image. (**b**) The multi-scale Top-hat transform. (**c**) the multi-saliency fusion detection.

The size of the first kind of infrared image is 1024×768, and the image has two targets, namely, aircraft and bird. The original infrared image is shown in Figure 3a, processed by our algorithm. Firstly, it is processed by the multi-scale top-hat brightness transformation algorithm. The result is shown in Figure 3b. It is obvious that most of the background is effectively suppressed and small noise points remain, but the target is well preserved, and the brightness is complete and the boundary is clear. Then, the final target saliency map is obtained by multi-saliency fusion detection, as shown in Figure 3c. The background noise is almost completely suppressed, and only two targets remain. The saliency of the large target aircraft is good, there is no blurred boundary and incomplete, and the saliency detection is complete; the small target is not omitted and prominent.

**Figure 4:**
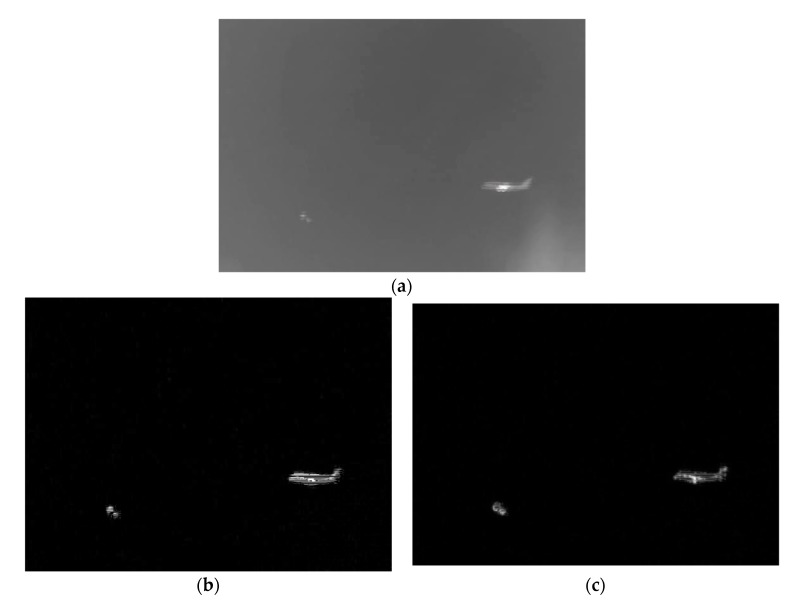
The saliency detection results of this algorithm for the second kind of infrared image. (**a**) The original image. (**b**) The multi-scale Top-hat transform. (**c**) the multi-saliency fusion detection.

The size of the second kind of infrared image is 580×432, and the image also has two targets, namely, aircraft and bird. The original infrared image is shown in Figure 4a. The whole image is very fuzzy, and there are some clouds in the background. The two targets of the airplane and the bird and are very similar to the background. Besides, only the middle part of the plane is bright, and the bird is very weak. Then, the result of the original infrared image processed by the multi-scale Top-hat transform in this paper is shown in Figure 4b. In Figure 4b, the aircraft and the bird are well preserved, most of the cloud background is suppressed, but there are a few white speckle noises. Finally, the result of our algorithm is as shown in Figure 4c. Not only the background of the image is very clean, but also the two targets of the aircraft and the bird are very prominent, almost consistent with the original image.

**Figure 5:**
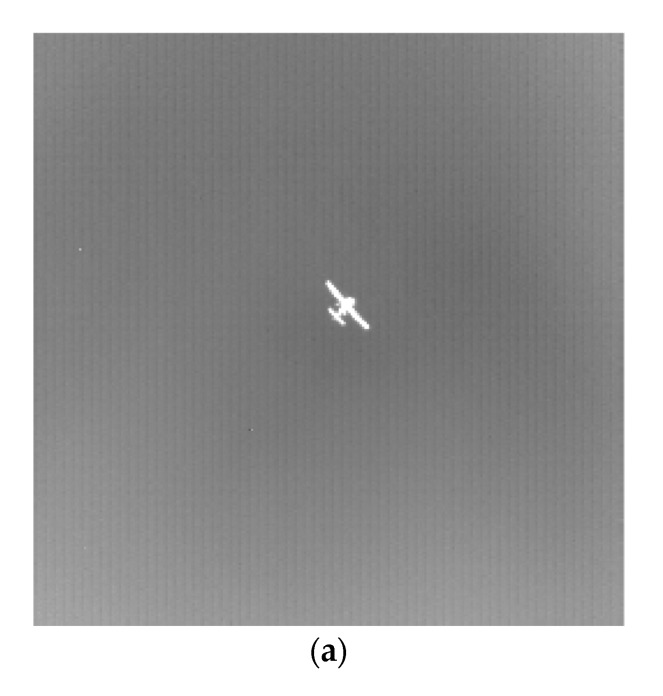

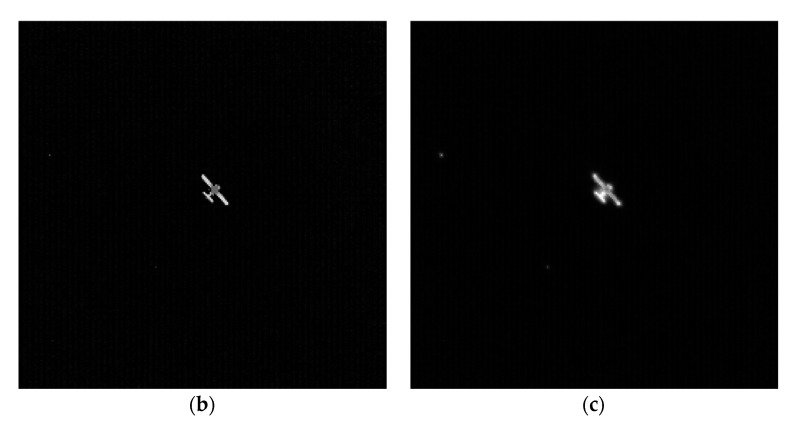
The saliency detection results of this algorithm for the third kind of infrared image. (**a**) The original image. (**b**) The multi-scale Top-hat transform. (**c**) the multi-saliency fusion detection.

The size of the third kind of infrared image [16] is 256×256, and there are three targets in the image: two highlights and the aircraft. The original infrared image is shown in Figure 5a. The background of the image is fuzzy, the two bright spots are very weak, and the aircraft target is relatively bright and large. After multi-scale top hat transformation, the results are shown in Figure 5b. Two bright spots were retained, but weaker. In contrast, the aircraft is bright and complete. Besides, the image background is suppressed, but there is a lot of strip noise. Finally, the result of our algorithm is obtained through multi-scale significance fusion detection, as shown in Figure 5c, the background is very clean, the two bright small targets are salient and the aircraft is more prominent.

After the above three types of infrared image tests, it is shown that the saliency detection of our algorithm is suitable for infrared multi-target saliency detection under blurred sky background conditions.

### 3.2. Comparision and Analysis of Various Algorithms

To further analyze the performance of the proposed algorithm and the improvement of target saliency detection, five different saliency detection algorithms and the proposed algorithm are firstly used to process the same infrared target image, and then the advantages and disadvantages of the saliency detection of various methods are compared and evaluated. The used five classic saliency detection algorithms are ITTI, SEG [17], GBVS, SUN [18], and TOP-HAT [19]. For the convenience of describing the algorithm in this paper, it is named Multi-Target (MT). The processed data are unified into infrared target images taken by an infrared thermal imager in the sky background.

**Figure 6:**
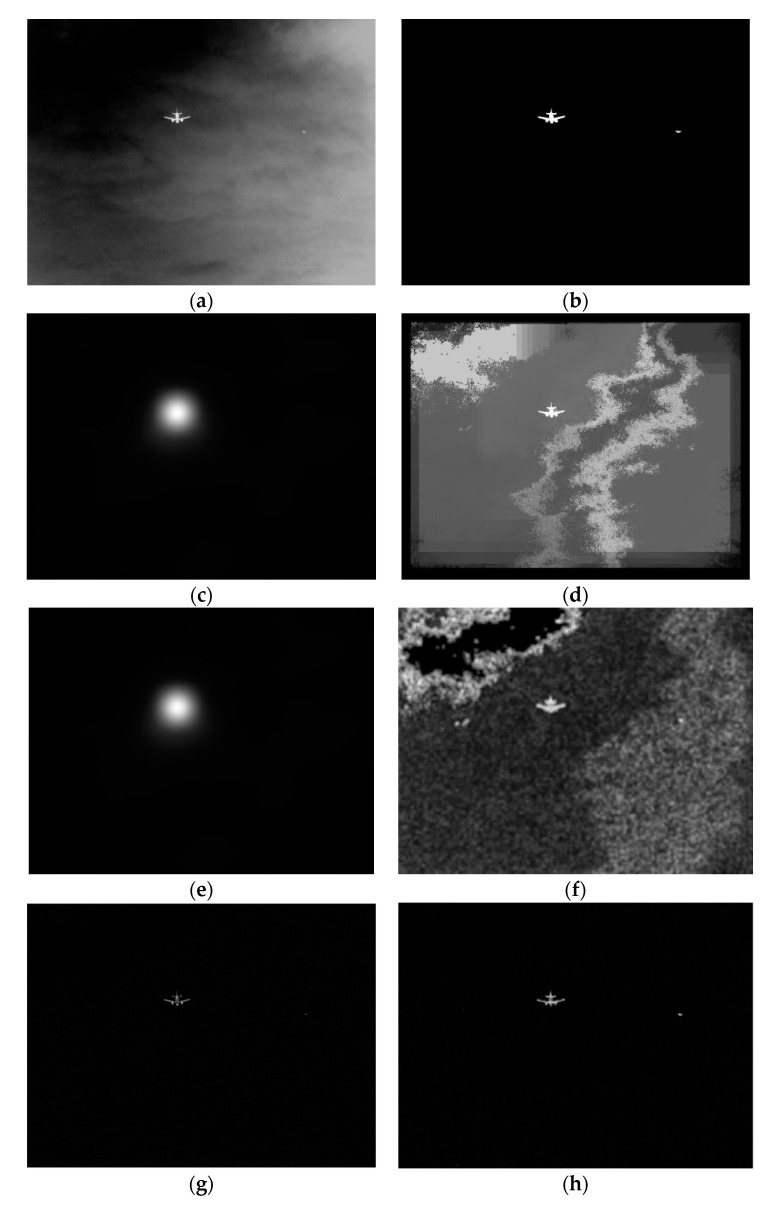
Comparison of saliency detection between the proposed algorithm and five saliency detection algorithms. (**a**) Original map. (**b**) Label map. (**c**) ITTI. (**d**) SEG. (**e**) GBVS. (**f**) SUN. (**g**) TOP-HAT (**h**) MT.

In Figure 6, Figure 6a is the original map, the size of the image is 1024×768, and it includes two targets: aircraft and bird. Figure 6b is the label map after marking two targets in the original map. Figure 6c,e are the saliency detection maps of the ITTI and GBVS algorithms, respectively. The processing results of the two algorithms are similar. The saliency maps of the larger target aircraft are bright and complete, and there is no defect, but the outline of the target is very unclear, showing the shape of a ball. The shape of the target is too different from that of the target, and the two algorithms have lost the small and weak target bird in the sky, and have not prominently highlighted the small and weak target. Figure 6d,f are the saliency detection maps of the SEG and SUN algorithms, respectively. The two algorithms have a better effect on saliency detection of targets. They can detect two targets with a clear outline, but the effect of background suppression is too poor, and there are a lot of noisy backgrounds not filtered out. Figure 6g is the saliency detection maps of TOP-HAT. Its saliency detection effect is better on the whole. It can clearly detect the two targets, plane and bird, and suppress most of the background, but there are still many points of noise, showing salt and pepper shape. Figure 6h is the saliency detection map of MT algorithm. The saliency detection effect of MT algorithm is better than those of the above five algorithms. The two targets are clear and visible. It not only has better integrity and clear outline but also has better background noise suppression effect. It shows that MT algorithm has a great superiority in infrared multi-target detection under the ambiguous background of the sky.

**Figure 7:**
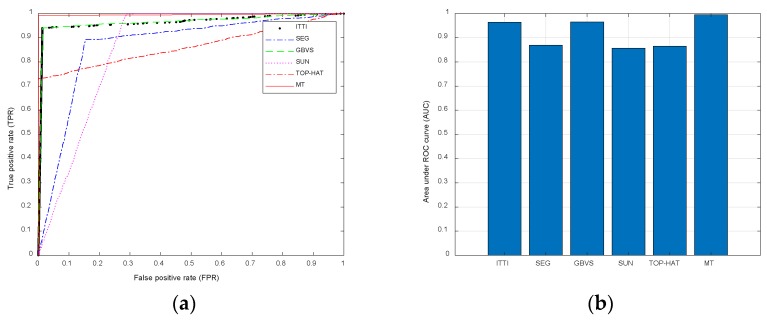
Comparisons between the proposed algorithm and five algorithms. (**a**) ROC curves of different algorithms. (**b**) AUC values of different algorithms.

To further compare the performance of MT algorithm with the other five saliency detection algorithms, the true positive rate TPR and false positive rate FPR are used to generate the ROC curve [20], and the area AUC under the ROC curve is calculated to generate a cylindrical graph. The calculation of ROC parameters is as follows:

(28) TPR=TP/(TP+FN)

(29) FPR=FP/(FP+TN)

where the true Positive TP is the part of the real target in the target salient area, the false positive FP is the part of the target salient area that is not the real target and the error salient area, the true negative TN is the part of the whole image that removes the salient area of the target and the real target area is not salient, the false negative FN is the part of the real target that is not salient, as shown in Figure 7a. Therefore, the closer the ROC curve is to the upper left corner, the larger the AUC value of the area under the ROC, the better the salience effect of the algorithm. From Figure 7a, we can see that the ROC curves of ITTI and GBVS algorithms are close, and the salience effects of the two algorithms are similar; the ROC curves of SEG algorithm are far from the upper left corner, and the salience effect is poor; the ROC curves of SUN algorithm deviate most from the left side, the ROC curves of TOP-HAT algorithm deviate most from the upper side, and their salience effect is worse. Compared with the above five algorithms, the ROC curve of MT algorithm is the highest and near the upper left, which shows that the performance of the algorithm is better than the other five algorithms and the effect is better. Then the AUC values of different algorithms are compared. In Figure 7b, the height of SUN, SEG and TOP-HAT algorithms is similar and relative low, AUC values are 0.8571, 0.8691 and 0.8654, respectively. The height of ITTI and GBVS algorithms is higher and similar, AUC values are 0.9648 and 0.9658, respectively, which are better than SUN and SEG. MT algorithm is the highest, the AUC value is 0.9957, with salience effect best. It is a complete and accurate way to highlight the target. The above two evaluation results are the same, and it is concluded that the proposed algorithm is superior to the other five saliency detection algorithms, which improves the effectiveness of multi-target saliency detection in the sky blurred background.

To further compare MT algorithm with the other five algorithms in terms of efficiency, different algorithms are used to process the same frame in the three types of infrared images. The corresponding frames in the three types of infrared images are Figure 3a, Figure 4a and Figure 5a respectively, and the sizes are 1024×768, 580×432 and 256×256, respectively. The running time results of the algorithm are shown in Table 1. Our algorithm runs much faster than SEG and SUN, and its efficiency is better than SEG and SUN. Although the MT algorithm is slower than ITTI, GBVS, and TOP-HAT, the difference is small, and with the size of the infrared image becoming smaller, the MT algorithm runs a 1.43 s per frame.

Combining all the above experimental results, it can be concluded that MT algorithm is superior to the other five algorithms in terms of significance detection, the background suppression effect of the generated saliency map is better, the targets in the map are very prominent and complete, and the running efficiency is also very fast, so the MT algorithm is suitable for multi-target saliency detection in a sky blurred background.

## 4. Conclusions

This paper mainly improves and proposes a saliency detection algorithm for infrared images, aiming at solving the issues of multi-target infrared images in a blurred sky background. Firstly, infrared images are preprocessed, and different size templates are used to suppress the background information through multi-scale Top-hat light and dark suppression, and different sized targets are retained to obtain multi-target image after background suppression. Then, a saliency detection method based on the fusion of three target features, namely, spectrum residual, phase spectrum and quaternion Fourier transform, is proposed. Firstly, two saliency detection methods, spectral residual and phase spectrum, are improved by a multi-scale fusion method to generate a multi-scale saliency map. Then, the two saliency maps of infrared images are fused with the saliency detection map of the quaternion Fourier transform to get the final saliency map of the infrared image. The saliency map generated by this algorithm overcomes the image blurred background. When the target is hidden in the background, multiple targets of different sizes are detected at the same time. The target is not incomplete, the boundary is clear, and the effect of background noise suppression is good. The main disadvantage is that it is impossible to detect the target in the infrared image behind the cloud background, and the saliency detection can not separate the target when the target overlaps.

## Figures and Tables

**Table 1:** The running time of different algorithms for processing the same one frame.

Pixels	ITTI	SEG	GBVS	SUN	TOP-HAT	MT
1024 × 768	0.60 s	67.34 s	2.56 s	66.33 s	0.80 s	9.37 s
580 × 432	0.52 s	30.95 s	2.35 s	20.90 s	0.28 s	3.55 s
256 × 256	0.35 s	16.35 s	2.12 s	5.91 s	0.10 s	1.43 s
